# Offloading and Transmission Strategies for IoT Edge Devices and Networks

**DOI:** 10.3390/s19040835

**Published:** 2019-02-18

**Authors:** Jiheon Kang, Doo-Seop Eom

**Affiliations:** School of Electrical Engineering, Korea University, Seoul 02841, Korea; kanghead@korea.ac.kr

**Keywords:** deep learning, edge computing, internet of things, offloading

## Abstract

We present a machine and deep learning method to offload trained deep learning model and transmit packets efficiently on resource-constrained internet of things (IoT) edge devices and networks. Recently, the types of IoT devices have become diverse and the volume of data has been increasing, such as images, voice, and time-series sensory signals generated by various devices. However, transmitting large amounts of data to a server or cloud becomes expensive owing to limited bandwidth, and leads to latency for time-sensitive operations. Therefore, we propose a novel offloading and transmission policy considering energy-efficiency, execution time, and the number of generated packets for resource-constrained IoT edge devices that run a deep learning model and a reinforcement learning method to find an optimal contention window size for effective channel access using a contention-based medium access control (MAC) protocol. A Reinforcement learning is used to improve the performance of the applied MAC protocol. Our proposed method determines the offload and transmission strategies that are better to directly send fragmented packets of raw data or to send the extracted feature vector or the final output of deep learning networks, considering the operation performance and power consumption of the resource-constrained microprocessor, as well as the power consumption of the radio transceiver and latency for transmitting the all the generated packets. In the performance evaluation, we measured the performance parameters of ARM Cortex-M4 and Cortex-M7 processors for the network simulation. The evaluation results show that our proposed adaptive channel access and learning-based offload and transmission methods outperform conventional role-based channel access schemes. They transmit packets of raw data and are effective for IoT edge devices and network protocols.

## 1. Introduction

In recent years, the number of internet of things (IoT) applications and products has been increasing in the home, medical, industrial, and military fields to sense and to control environmental events [1]. In general, the data generated by IoT edge devices such as sensors and actuators are transmitted to cloud servers via wireless communications (e.g., Wi-Fi, bluetooth low energy (BLE), or long range wide area network (LoRaWAN)), and the collected data are processed or analyzed in the cloud. However, transmitting large amounts of raw data such as video, images, and voice to the cloud is expensive for the following reasons [2,3]. First, the time delay or latency caused by limited bandwidth and unstable channel conditions (e.g., congestion, interference, and collisions), leads to slowed decision making for time-sensitive operations. Second, centralized cloud centers are inefficient and expensive for performing data processing on the large amounts of collected data from various types of IoT devices, because of supporting various processing methods, and the necessity of servers and storage expansion. To overcome these disadvantages of the traditional cloud computing structure, cloud centers have been placed closer to the network edge, thereby reducing the communications bandwidth and amount of traffic required between the edge devices and the cloud center by handling the data nearby the source of generated data [4].

Edge computing located at the network “edge” is a key technology for IoT services such as time-sensitive and resource-constrained applications [5]. Because edge computing provides faster responses, and computer nodes are distributed at each edge network, the total traffic flows, bandwidth requirements, and transmission latency are reduced, as well as allowing the offloading of computational overhead compared to the centralized cloud computing structure [6]. Edge computing is able to offload network and computing resources to improve the transmission efficiency and resource utilization; however, transmission failures and delays due to congestion and interference on the edge layer (i.e., the connection between the edge server and edge devices) are still challenging problems [7].

Deep and machine learning approaches have been introduced into IoT applications for high efficiency in big and complex data [8,9,10]. Deep learning architectures usually have many layers and neurons that require much memory and computation to extract nonlinear feature vectors and predict outputs with high accuracy. Indeed, most of the IoT services that applied deep learning models for analyzing and processing the collected data from IoT devices are performed in the cloud with high-performance resources. Edge computing and in-device computing lead to reduction of required communication overhead to reach the cloud [11].

Recently, some studies have been conducted to apply trained deep learning models to resource-constrained IoT edge devices by optimization techniques such as fixed-point quantization [12,13], network pruning [14], and hardware/software acceleration [15,16], for cases when the IoT devices are not always connected to the network. The actions are performed more accurately by deep learning processing than by traditional signal processing or applied machine learning methods. In addition, a transmission scheduling method for offloading is proposed to select an optimal scaled-down size of the original data, considering network capacity and using shared deep neural network (DNN) models, designed with fewer neurons in the upper hidden layer than the lower layer, on the IoT edge devices and server [17].

Undoubtedly, deep learning has been a state-of-the-art solution in many areas such as classification and regression domains (e.g., image, video, and natural language processing), even though it is not always possible to obtain optimal results [11]. In particular, when migrating a trained deep learning model to a resource-constrained micro controller unit (MCU), such as those commonly used in IoT edge devices, it is important to consider latency and energy efficiency in determining whether to send the fragmented packets of raw data or transmit the output vectors of a deep learning network. For example, if the amount of data to be transmitted from the edge device to the edge server is small or the communication channel is idle, directly sending the packets results in less energy consumption and low latency. On the contrary, if the edge device needs to send a large number of fragmented packets of data under heavy congestion or interference, sending compressed data or the output result of a deep learning network may be effective in terms of channel utilization and improving the transmission success ratio.

Thus, we introduce our novel offloading and transmission strategy using deep and machine learning for IoT edge devices and networks to improve the classification accuracy of sensory data, as well as the network performance and energy efficiency. Our system consists of three steps. In the first step, each edge device estimates the average latency and the average transmission success ratio required to transmit a packet to the edge server though communication channel monitoring based on Q-learning, which is a reinforcement learning method. Reinforcement learning is applied to improve the general performance of MAC protocol. In the second step, each IoT edge device calculates the cost for transmitting the measured raw data or the output feature of the deep learning model using the measured average latency and transmission success ratio, as well as the operation performance and the power consumption. The expected latency and power consumption are computed based on the execution time for each layer of the applied deep learning structure and the intermediate output data size of the corresponding layer. The number of fragmented packets of the intermediate output data is calculated to estimate the expected latency and power consumption for transmitting the total data to the edge server. Finally, the edge device transmits the raw data or intermediate output data or final output data to the edge server, according to our proposed offload and transmission strategy with minimum latency and power consumption.

**Figure 1:**
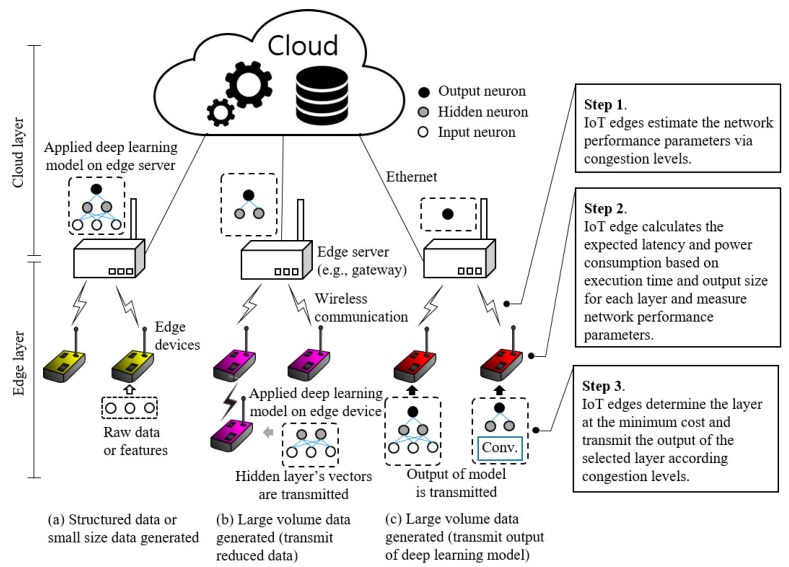
Offload and transmission strategy overview based on generated data size and computation performance considering media channel condition on IoT edge device with deep learning model.

Figure 1 presents our proposed offload and transmission scenarios based on a shared deep learning model for IoT edge devices and edge servers (e.g., gateway, access point, and light-weight server machine). In case the data measured at the edge device are structured data such as temperature and humidity, or the extracted feature data by traditional signal processing methods or raw data are smaller than the length of the application payload in packet data units (PDUs), directly transmitting the measured raw data without any deep learning processing may be effective. Otherwise, if the edge device generates a relatively large volume of data such as image, video, and sensory signals, the edge device should determine whether to send fragmented packets of the total data frame or send output data through deep learning processing. Depending on the expected latency and power consumption, the intermediate data of the hidden layer or the output data of a deep learning model is transmitted. To determine the transmission cost, we consider the power consumption of the transceiver and the microprocessor, the computation time of the microprocessor, and the expected latency to send all the fragmented packets. The key contributions of our study are summarized as follows:

1. We provide a novel deep learning approach for IoT edge devices and networks to transmit measured data to edge servers considering the network performance as well as the capacity of resource-constrained microprocessors.
2. We apply reinforcement learning based on Q-learning to learn the optimal backoff scheme in the contention-based MAC protocol to improve the network channel utilization considering the current channel condition (e.g., four states: idle, low, high, and burst traffic).
3. Our proposed offload and transmission strategies can handle the different rates of data flow and load of the nature of IoT applications.
4. We implemented a deep learning model on a low-power and performance Cortex M7 (216 MHz and 120 MHz) and Cortex M4 (80 MHz) microprocessor and measured the operation time and power consumption for each layer of the deep learning model. In addition, we used the measured performance metrics in a simulation and verified that our proposed methods can be applied to actual IoT edge networks through experiments.

Compared to following predefined roles, our proposed the optimal backoff scheme for the contention-based MAC protocol and the offload and transmission strategy are an effective and adaptive method for learning the current state of the channel and the computation performance of target devices. 

The remainder of this paper is organized as follows: Section 2 discusses related works of deep learning for IoT edge devices and networks. Section 3 describes the proposed optimal backoff scheme to improve the channel utilization. Section 4 describes the proposed offload and transmission strategy. Section 5 summarizes the performance of our proposed methods. Finally, Section 6 summarizes and concludes the paper.

## 2. Related Works

We first introduce the applicability and efficiency of machine and deep learning in terms of IoT edge devices and their applied network protocols, and then we discuss the differences in our work compared to previous studies.

### 2.1. Deep Learning for IoT Edge Devices

Deep learning architectures can effectively extract the feature of sensory data (e.g., images, voice, and time-series) and classify the desired output for diverse IoT applications. Convolutional neural network (CNN)-based image classification showed state-of-the-art performance. In addition, recurrent neural network (RNN)-based deep learning structures showed that they could process data effectively compared to conventional signal processing methods and traditional machine learning methods. Based on these achievements, studies that analyze the data measured and collected from sensors using deep learning are increasing, as well as image, video, and natural language processing.

In [18], CNNs have successfully used sensory signals for electrocardiogram (ECG) classification and anomaly detection. Kang et al. [19] introduced vibration sensor-based structural health monitoring and an early fault detection system by an ensemble deep learning model. In addition, hybrid CNN-RNN models are widely used with time-series sensory signals such as human activity recognition [20] and stock price estimation [21]. However, the applications mentioned above all are performed on high-performance computational machines in both an offline phase for training and an online phase for execution. Furthermore, as the size of a deep learning model increases for improving performance, the memory requirement also increases significantly. 

Han et al. [14] and Iandola et al. [22] reported that a trained deep learning model could be applied to embedded devices by network pruning with quantization (less than 8 bit) and Huffman encoding with a combination of 1 × 1 convolutional filter. Most of the literature on enabling deep learning on IoT edge devices also employs pruning and quantization methods to reduce the memory utilization and specifically designed software and hardware accelerators to speed up the operation [13,23]. Du et al. [24] also proposed a streaming data flow to achieve higher peak throughput and greater energy efficiency for CNN acceleration architectures for IoT devices. These methods allow minimizing the loss of accuracy when applying a deep learning model on a resource-constrained device. Because diagnosis and surveillance applications on IoT environments have often demanded high accuracy and real-time requirements, an optimized and trained deep learning model should be carefully considered to achieve results within a limited processing time and with acceptable accuracy on resource-constrained IoT devices. Additional details of distributed deep learning applied to IoT devices, networks, and applications are available in [11].

### 2.2. Deep Learning for IoT Edge Networks

In IoT, a number of edge devices such as sensors and actuators co-operate to transmit data considering the energy consumption, latency, and packet error rate. The edge devices used in typical IoT applications consume most of their energy in transmitting and idle time [25]. Therefore, efficient channel access and scheduling methods such as the MAC protocol, which can decrease the latency and increase the fairness and transmission ratio, are required. 

Liu et al. [26] introduced RL-MAC, which estimates an adaptive duty-cycle and transmission active time based on the traffic load and channel bandwidth by reinforcement learning. In [27], a QL-MAC with Q-learning is proposed, whereby the sleep and wakeup scheduling is adaptable depending on the network traffic load. The modified protocol [28] is targeted to vehicle-to-vehicle communication based on IEEE 802.11p MAC, and Q-learning is applied to select the optimal contention window (CW) size to reduce the packet collision probability. 

Li et al. [17] designed a novel offload scheduling method to optimize the network performance of deep learning-based applications in edge computing. Their proposed scheduling algorithm attempts to assign the maximum number of deep learning tasks to both the edge devices and edge servers with corresponding deep learning layers, considering the service capacity and network bandwidth. Their proposed method is similar to our work, in that it considers the processing time and the output data size of the intermediate layer of the deployed deep learning model on edge devices. However, their proposed method only utilizes the known service capacity and the maximum available bandwidth, and possible side effects due to collisions and interference are not considered. Considering the current network conditions is required for a more effective offload and transmission strategy.

### 2.3. Novelty of Our Work Compared To Related Works

In this section, we summarize the differences in our work compared to other studies. Although we applied a well-known quantization method that represents a 32-bit floating-point as an 8-bit fixed-point to operate the trained deep learning model on resource-constrained IoT edge devices [29], our proposed method is the first offloading approach in the IoT edge layer that considers the output size, execution time, and power consumption of each layer of the deep learning model on resource-constrained microprocessors operating at 216 MHz or less. 

In addition, our proposed novel offloading and transmission strategy chooses among three cases, either sending the raw data directly, or the desired output, or the intermediate output data of the deep learning model, in the most efficient way to reduce the energy consumption and latency considering the current network status. The transmission cost for each case is computed as a weighted sum of the required latency and power consumption for transmitting the packets as well as the execution time and power consumption for the deep learning processing. 

In particular, our proposed transmission scheme can be applied widely to systems that can estimate the average latency and transmission success ratio by channel or packet monitoring.

## 3. Reinforcement Learning-Based MAC Protocol with an Adaptive Channel Access Scheme

In this section, we introduce our proposed adaptive contention-based MAC protocol with a backoff scheme that can estimate the optimal CW size using Q-learning. We employed the concepts of a well-defined Q-learning-based MAC protocol [26,27,28], and then we redefined the states and action space as well as the reward function and Q-function according to the channel conditions (i.e., idle, low, high, and burst traffic). We proposed a reinforcement learning-based adaptive channel access scheme to improve the performance of the MAC protocol before applying an offload and transmission strategy. 

### 3.1. Q-Learning

Q-learning is one of the most popular and powerful reinforcement learning algorithms, the goal of which is to obtain the optimal policy of a sequence of actions that maximizes the accumulated reward in an unknown and model-free environment [30]. Owing to the difficulty in accurately recognizing the channel environment and designing a communication model considering collisions, padding, and interference in wireless communications, we employed Q-learning, which is a well-known off-policy temporal-difference algorithm, for self-learning in IoT edge devices. The Q-function indicates the optimal action at a corresponding state. The Q-values of the state–action pair (*s_t_*, *a_t_*) are updated as follows:

(1) $$\begin{array}{c} \hat{Q}\left(s_{t},a_{t}\right)\leftarrow \left(1-\alpha \right)\hat{Q}\left(s_{t},a_{t}\right)+\alpha \left(r_{t+1}+\gamma \underset{a_{t+1}}{\max}\hat{Q}\left(s_{t+1},a_{t+1}\right)\right), \end{array}$$

where $\hat{Q}$ denotes the learner’s current approximation to Q, *α* ∈ (0,1] is the learning rate, and γ∈[0,1] is the discount factor and has the effect of valuing rewards received earlier higher than those received later. *s*, *a*, and *r* represent states, actions, and reward, respectively, and these are set up for the proposed methods.

### 3.2. Estimating optimal Contention Window Size

One of the main goals of a contention-based MAC protocol is to avoid packet collisions. Packet collision occurs when multiple nodes simultaneously access a channel. Thus, the node must check whether the state of the channel is idle or busy to avoid collision. Generally, the clear channel assessment (CCA), provided by the RF transceiver, is used to check the channel status. A backoff mechanism is required to transmit after a certain delay when the medium channel state is busy. Determining the backoff duration requires a CW and the channel access efficiency is determined by how well the CW size is selected. Adaptive CW selection algorithms are most commonly used in carrier-sense multiple access with collision avoidance (CSMA/CA) to improve the throughput and fairness, and to reduce the latency and the collision probability in modern applications [31,32]. In general, a random backoff scheme within the CW is used for channel access. The duration of the backoff is randomly selected in the range of 0 to CW size. The CW increases owing to an increase in congestion, and a decrease in the CW is performed to access the channel more quickly owing to reduced congestion. Although designing an optimal channel access mechanism by modeling and monitoring all of the channel environments is difficult, a policy of selecting the appropriate CW considering the channel condition is essential. Thus, we adopted a Q-learning-based adaptive CW selection scheme, and defined a reward function to be maximized when the number of backoffs and access time is minimum to rapidly access the channel. The state space *s* contains CW sizes according to a binary exponential random backoff scheme for each congestion level. *ch* means the congestion level (i.e., idle, low, high, burst traffic) according to the generated amount of packet. The actions *a* determine the CW at *t* from CW at *t* − 1. The proposed reward function to minimize latency is as follows:

(2) $$\begin{array}{l} s[ch]\in \{3(CW_{\min}),4,5,6,7,8(CW_{\max})\},\\ CW_{t}=a\in \{CW_{\min},CW_{t-1}-1,CW_{t-1},CW_{t-1}+1,CW_{\max}\},\\ r_{t+1}=r\left(s{\left[ch\right]}_{t},a_{t}\right)\\ \;\;=\left\{ \begin{array}{l} \frac{CW_{\max}}{CW_{t}}\times \frac{macMaxCSMABackoffs}{N_{CCA}+1},\mathrm{if}\;\mathrm{transmitted}\\ 0,\;\mathrm{if}\;\mathrm{dropped},\;N_{CCA}>macMaxCSMABackoffs \end{array}\right.,\\ backofftime_{t}=random\_uniform(0,2^{CW_{t}}-1), \end{array}$$

where *s*[*ch*] means managing state *s*, used for the Q-learning by each of the four congestions at level *ch*. The values of *s* are predetermined from 3 (*CW*_min_) to 8 (*CW*_max_). The *CW* at *t* is determined by whether a previous time *t* − 1 value is held, incremented or decremented by one step, or set to minimum or maximum. The Q-learning agent selects a CW that maximizes reward in state *s*[*ch*] at *t*, where *N_CCA_* and *macMaxCSMABackoffs* denote the number of CCA counts and the maximum number, respectively. A higher reward suggests a lower number of CCA and lower backoff time. Finally, $backofftime_{t}$, which is the value of the backoff duration at *t*, is randomly selected in the range of 0 to $2^{CW_{t}}-1$.


**Algorithm 1: Q-learning-based Backoff Mechanism**
1**Initialization**: 2  *N**_CCA_*** = 0, *load_Q_table*() // load a previously learned value3**Input**: medium channel state by CCA (idle or busy), packet transmission (success of failure) 4**Output**: backoff duration (*backofftime*)5**while** mac_protocol **do**6 **if** active_mode 7  *busy_count* += CCA(*is_busy*) or *busy_count* −= CCA(*is_idle*)8  // channel mode is set to 4 levels according to busy_count9  *ch_mode* = *channel_mode*(*busy_count*);10 **end if**11 **if**
*event_send_request*12  **while**
*N_CCA_* < *macMaxCSMABackoffs* or *packet_transmitted*13   // set the largest value in the Q table to next CW14   *CW_t_* = *Max_Q_table(ch_mode, CW_t − 1_)*15   *Backoff*(*backofftime* = *uniform_random*(0, 2^CWt^ − 1) )16   **if**
*CCA*(*is_busy*)17    *busy_count* += 1; *N_CCA_* += 1; 18   **else if**
*CCA*(*is_idle*)19    *transmit*(*packet*) and *wait*(*Ack*)20    *busy_count* −= 1; *N_CCA_* = 0;21   **end if**22   *ch_mode* = *channel_mode(busy_count)*23   **if**
*learning_mode*24    Q(*s_t_*[*ch_mode*]: *CW_t_*_ − 1_, *a_t_*: *CW_t_*) - Equation(2)25   **end if**26    // network parameter measurement27    *transmission_success_ratio (*$r_{t}$) = # of received_ack / # of requested packet,28    *average_latency* ($l_{a}$) = *ack_time* – *transmit_time* (about only transmitted packet)29    // other parameters else (e.g., *meanBackoffduration*, *channelAccessratio*, etc.)30  **end while**31  *delete_queue*(*packet*) // transmitted or dropped32 **end if**33
**end while**


Algorithm 1 shows the mechanism of the proposed Q-learning-based backoff scheme to effectively access the medium. The IoT edge device periodically monitors the channel during the active mode and classifies the result into four levels based on the value of *busy_count* in a short period of time (e.g., 10 measurements over 500 ms). When a packet transmission event is requested, and *N_CCA_* is less than *macMaxCSMABackoffs*, the backoff is performed by selecting the *CW_t_* considering the current channel state based on the maximum Q-value in the learned Q-table. After a delay of the backoff time, CCA is performed to verify that the channel is available. When the channel is busy, the corresponding parameters, *busy_count* and *N_CCA_*, are incremented. In the other case, the method sends the packet and waits for an *Ack* frame to be received, upon which *busy_count* is decremented and *N_CCA_* is initialized. If in learning mode, the Q-value of the current channel state is updated by the reward function. The network performance indicators (e.g., transmission success ratio, average latency, and mean backoff duration) are updated based on the result of packet transmission. The measured network performance parameters are used in Section 4 to calculate the average number of retransmission counts ($m_{r}$) and the expected latency ($t_{c}$) required to successfully send a packet to its destination. Our proposed learning-based method can be widely applied to contention-based MAC protocols.

## 4. Offloading and Transmission Strategy

**Figure 2:**
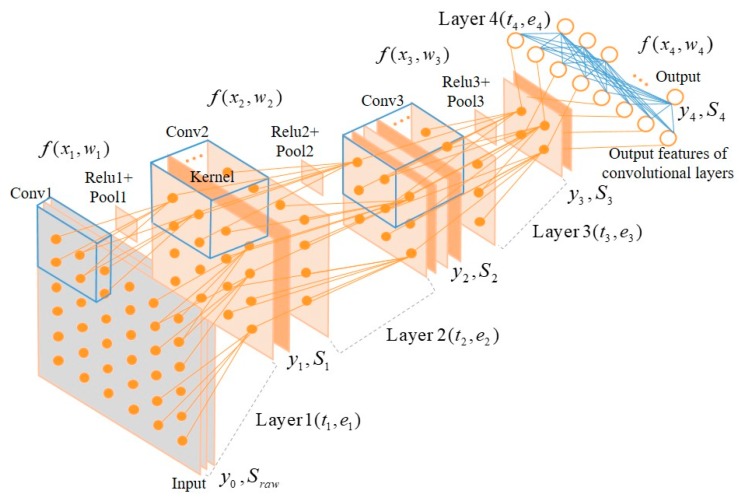
Applied deep learning structure (i.e., convolutional neural network). Blue cubes and solid lines present the learnable parameters.

In this section, we introduce a novel offload-based transmission strategy that considers energy efficiency and delays in the IoT edge layer, based on the improved MAC protocol, which is the method proposed in the previous section. We applied the quantization method to migrate the trained deep learning model to resource-constrained IoT edge devices [29]. We already know the learnable and hyper-parameters as well as the input data vector of each layer of the deep learning model, as shown in Figure 2. 

Therefore, we can calculate the execution time based on the system clock of the target microprocessor and output vector size of the next layer by computing the previous layer’s input data and weights, and the power consumption can also be calculated or measured during the operation. The related parameters of the deep learning networks used in our proposed offload and transmission strategy are given by the following expressions:

(3) $$\begin{array}{l} y_{l}\underset{t_{l},e_{l}}{=}f\left(x_{l},w_{l}\right),\\ T_{n}=t_{1}+t_{2}+\ldots +t_{n-1}+t_{n}=\sum_{l=1}^{n}t_{l},\\ E_{n}=e_{1}+e_{2}+\ldots +e_{n-1}+e_{n}=\sum_{l=1}^{n}e_{l},\\ y_{l}=x_{l+1},\\ S_{l}=fragmention(y_{l}), \end{array}$$

where $x_{l}$, $w_{l}$, and $y_{l}$ denote the input, weight, and output vector in layer *l*, respectively. $y_{l}$ also indicates the input of the next layer *l* + 1. $t_{l}$ and $e_{l}$ represent the execution time and power consumption to compute *f*(*x_l_,w_l_*), respectively. *f*(*x_l_,w_l_*) includes all operations such as convolution, activation, and downsampling to extract the output vector for the next layer; $T_{n}$ and $E_{n}$ represent the total execution time and power consumption up to layer *n*, respectively; and $S_{l}$ is the number of packets, $y_{l}$ is fragmented into packets by the PDU size of the corresponding radio transceiver with the *fragmentation*() function. Figure 2 shows an input layer, three convolutional with activation and down-sampling operation layers, with a fully connected output layer. The execution time and output vector size for each layer except the input layer can be calculated based on the corresponding deep learning model and the performance of target microprocessor. Refer to Table 2 for the number of inputs, outputs, and execution time for each layer.

In addition, we estimated the expected cost to successfully transmit a packet to the destination such as an edge server or the next hop using our proposed learning-based MAC protocol. As mentioned previous sections, we measured the average number of retransmission counts ($m_{r}=1/r_{t}$) based on the transmission success ratio ($r_{t}$) and the average latency (*l_a_*) needed to transmit one packet from an IoT edge device to the server according to the channel state. We used the average retransmission counts and the average latency to define the expected latency ($t_{c}=m_{r}\times l_{a}$) required to successfully send a packet to the destination.

We designed a cost function to select the optimal strategy in terms of minimizing the latency and power consumption as follows:

(4) $$\begin{array}{l} {\mathrm{Cost}}_{raw}=\alpha \left(S_{raw}\cdot t_{c}\right)+\beta \left(S_{raw}\cdot m_{r}\cdot Tx_{p}\right),\\ {\mathrm{Cost}}_{offload}=\alpha \underset{\mathrm{latency}}{\underset{︸}{\left(T_{n}+S_{n}\cdot t_{c}\right)}}+\beta \underset{\mathrm{power}\;\mathrm{consumption}}{\underset{︸}{\left(E_{n}+S_{n}\cdot m_{r}\cdot Tx_{p}\right)}}\\ (\mathrm{when}\;n=0,\;S_{0}=\mathrm{fragmentation}(y_{0}=x_{1})=S_{raw},\;T_{0}\;\mathrm{and}\;E_{0}=0),\\ {\mathrm{Strategy}}_{offload}=\underset{n\in [0,N]}{\mathrm{argmin}}\left({\mathrm{Cost}}_{offload}\right), \end{array}$$

Here, *α* and *β* are weight factors for the latency and power consumption, respectively, and *S_raw_* is the number of fragmented packets of measured raw data according to the PDU size. Cost*_raw_* represents the cost that is considered the latency (*S_raw_*·*t_c_*) and the energy consumption (*S_raw_*·*m_r_*·*Tx_p_*) required to transmit the *S_raw_* packets. *Tx_p_* is the transmission power of the radio transceiver. Cost*_offload_* represents the additional consideration of the execution time *T_n_* and power consumption *E_n_* when operating up to layer *n* of the applied deep learning model. Notice that when *n* = 0, *S*_0_ and *S_raw_* are the same. Using Strategy*_offload_*, we can find the optimal *n* parameter minimizing the transmission cost. In short, the edge device determines how many layers would be processed in terms of latency and energy efficiency. This means that the IoT edge device performs up to layer *n* and then transmits the corresponding output vectors, and the IoT edge server performs from layer *n* + 1 to the last layer *N*, considering the performance of the IoT edge device and the current channel state.

We did not fix *α* and *β*, the weight factors of latency and power consumption. Generally, transmission performance and energy efficiency is a trade-off. Therefore we designed the offload and transmission strategy to be configurable according to the priority of latency and power consumption when calculating the offload cost, Cost*_offload_*.

## 5. Experimental Results

### 5.1. Experimental Setup

In this section, we first describe the experiment settings for the learning-based MAC protocol and the offload and transmission strategy, and then discuss the evaluation results. In the experiments, we have two environments: one for network simulation, and another for executing the deep learning model on a resource-constrained IoT edge device. We designed the following experimental scenarios so that IoT edge devices can determine their offload based on the medium channel state and its computation performance: (i) The Q-learning-based adaptive channel access scheme was applied to improve MAC performance. (ii) We measured the network performance parameters (e.g., latency and transmission success ratio) according to each simulated congestion level. (iii) We measured and calculated an execution time, power consumption, and the number of output vectors for each layer of the deep learning model. (iv) Based on measured network performance parameters, operational performance of target devices, and the applied deep learning model, IoT edge devices selected which layer had the minimum cost for offloading and transmitting. 

**Figure 3:**
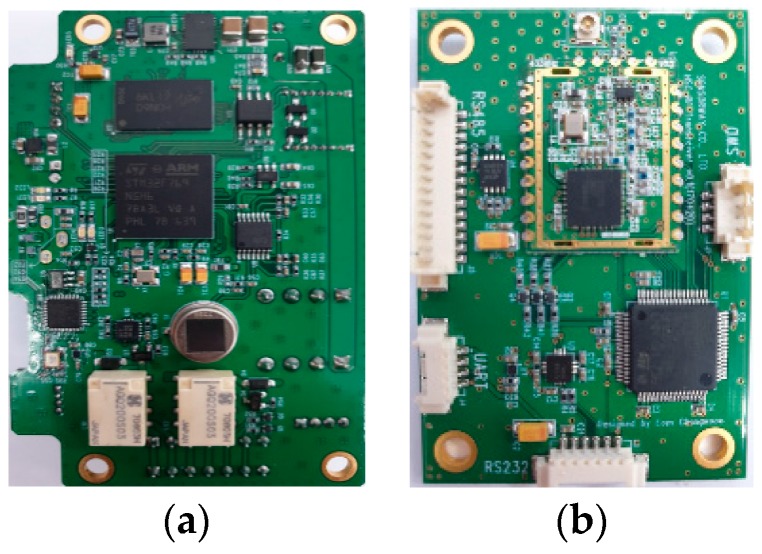
Applied deep learning structure (i.e., convolutional neural network). Blue cubes and solid lines present the learnable parameters. (**a**) STM32F769 board. (**b**) STM32L486 board.

To evaluate the performance of our proposed MAC protocol with the adaptive channel access scheme, we used nonslotted CSMA/CA of the IEEE 802.15.4 standard on OMNet++ (ver. 5.4.1) with the INET framework. We measured the runtime and power consumption for each layer of the applied deep learning model on a resource-constrained IoT edge device running at less than at 216 MHz (i.e., Arm Cortex-M7 (STM32F769) and Cortex-M4 (STM32L486)), and then applied the measured parameters to the network simulation and carried out our proposed offload and transmission strategy. In order to migrate the deep learning model learned on the back-end server to the IoT edge device, we used a quantization method to reduce the 32-bit floating-point weight and bias parameters to 8 bits fixed-point. A quantization method contributes in terms of memory efficiency and fast operation while minimizing the loss of the model accuracy. We used the CMSIS-NN kernel [29] for testing and measuring the performance on STM32F769 and STM32L486 embedded boards; Figure 3 shows our development boards. We used the MAX17201 stand-alone ModelGauge to measure the current consumption of the boards.

### 5.2. Performance Evaluation for Learning-Based MAC Protocol

**Figure 4:**
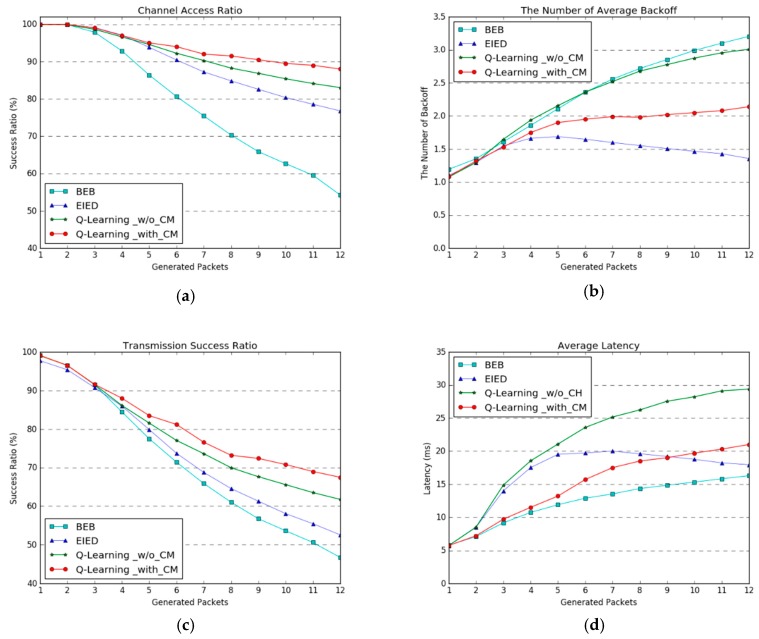
Experimental results for our proposed Q-learning-based adaptive channel access with channel monitoring method in comparison with other backoff methods: (**a**) Performance of channel access ratio, (**b**) number of average backoff for accessing the clear channel, (**c**) transmission success ratio, and (**d**) average latency when a packet is successfully transmitted. The horizontal axes of the graphs indicate the packets-per-second generated by a node at send interval, which represents the channel congestion level.

We performed the simulation and evaluation of our proposed learning-based MAC protocol with channel monitoring, and compared it with the binary exponential backoff (BEB), exponential increase exponential decrease (EIED), and Q-learning without channel monitoring protocols. Figure 4 illustrates the performance of the proposed scheme in comparison with the fixed-backoff mechanisms and without channel monitoring scheme. The vertical axis presents the performance metrics. The horizontal axis is the number of generated packets of length 112 bytes at the sending interval. The performance results plotted in Figure 4 are averages of 30 nodes, and all the experiments were performed without retransmissions. Table 1 shows the network simulation parameters.

Figure 4a shows the channel access ratio, which is the rate of attempted packet transmissions in the idle channel after the adaptive backoff time, and can be interpreted as the channel utilization. BEB had the lowest performance, by increasing the CW size step by step after initialization when there occurs channel congestion. The learning-based methods of selecting the adaptive CW were more effective than the fixed-backoff methods, and our proposed method of updating the Q-value for the corresponding channel state showed the best performance. The channel utilization and fairness were therefore improved by our method.

Figure 4b presents how many backoffs have to be performed to access an idle channel; it was not reflected in the results if the channel access failed. The average backoff count is the smallest when EIED is applied, because EIED allocates the maximum CW when the traffic load is increased. The reason why the backoff count is gradually decreased when the number of generated packets is more than four is that the number of nodes allocate the maximum CW owing to congestion. In the case of BEB, the CW is increased sequentially, and the average backoff count tends to increase as well. In the case of simple Q-learning without channel monitoring, selecting the next CW based on the previous CW does not reflect the channel congestion well. Learning based on the corresponding channel states is also effective in terms of the backoff count.

Figure 4c shows the transmission success ratio, which has a similar trend to the channel access ratio. This indicates that the transmission success ratio is improved by the number of channel access instances.

Figure 4d presents the average latency when a packet is successfully transmitted to the destination. BEB allocates a relatively short backoff time, which leads to congestion and decreases other performance metrics; however, it has low latency when the packet transmission is successful. When the transmission is unsuccessful, the average latency is measured in proportion to the increasing and decreasing tendency of the number of backoffs.

Using the simulation results, we estimated the average number of retransmission attempts required to successfully transmit a packet based on the average transmission success ratio. For example, if the transmission success ratio is 50%, the estimated number of retransmissions is 2. $t_{c}=m_{r}\times t_{r}$ defined in (4) can be obtained by using the average retransmission count and the average latency.

### 5.3. Performance Evaluation for Offload and Transmission Strategy

We carried out the performance of the proposed offload and transmission strategy using the average number of retransmission and the expected latency through the network simulation, and measured runtime and power consumption to execute migrated deep learning model on the resource-constrained IoT edge devices. We applied the deep learning model in Figure 2 to the STM32F769 and STM32L486 embedded boards; the parameters and the number of operations as well as the performance for each layer are shown in Table 2.

**Figure 5:**
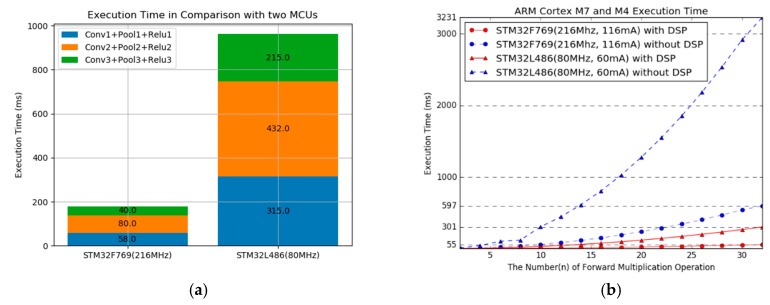
Execution time comparison with two boards used in experiment. (**a**) Execution time of the three convolutional layers of applied deep learning model. (**b**) Execution time by increases in the kernel depth of convolutional layer 1 according to the DSP unit.

Figure 5a illustrates a comparison of the execution time for each layer of the applied deep learning model on two IoT edge devices. The difference in the system clock is 2.7 times; however, the difference in the execution time is 5.4 times. As shown in Figure 5b, the increases in multiplication computation lead to a lager difference. We used ARM_MATH_CM4 and ARM_MATH_CM7 library to take advantage of the digital signal processor (DSP) unit in the Cortex-M4 and Cortex-M7 core, respectively. The performance results are shown in Figure 5b. As the results show, it would be difficult to apply our proposed offload and transmission scheme to IoT devices without the advantage of a DSP core. We measured power consumption for each board. The results were 60 mA and 116 mA, depending on clock speed. Current consumption for execution time and transmission power are reflected to calculate offload and transmission costs.

**Figure 6:**
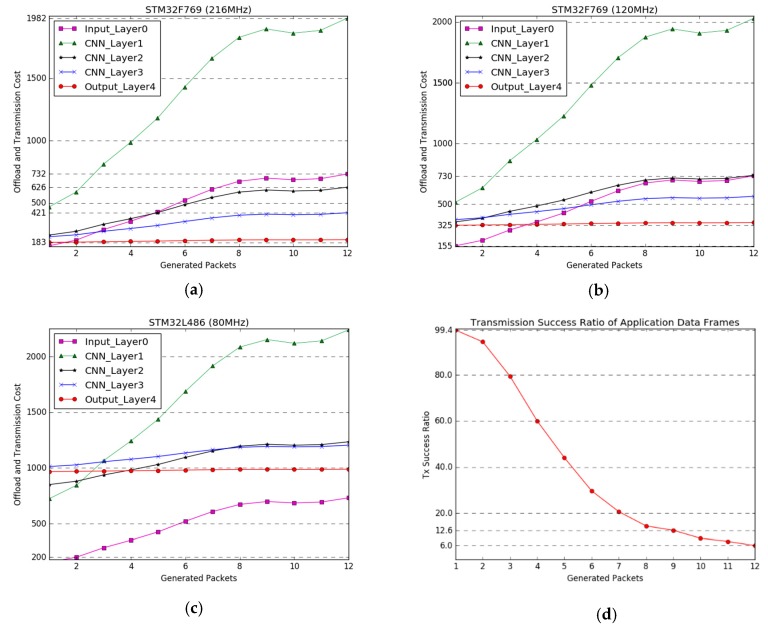
Experimental results for our proposed offload and transmission strategy based on the measured network performance metrics and deep learning structure in Figure 4 and its parameters in Table 2. (**a**–**c**) present the offload and transmission cost considering computation performance (operation clock speed), the number of packets generated in the corresponding layers, and media channel status. The horizontal axes of graphs (**a**–**c**) indicate packets-per-second generated by neighboring nodes at each send interval, representing the channel congestion level. (**d**) shows the transmission success ratio according to the number of fragmented packets generated in the application layer.

Figure 6a–c show the transmission cost in terms of the clock speed of the IoT edge devices and the number of fragmented packets corresponding to the output vectors of each layer of the applied deep learning model. The legends of the graphs indicate the number of fragmented packets of the size of the output vector for each layer according to the PDU size in Table 2. The horizontal axes of the graphs indicate the number of generated packets of 30 nodes, which represents the channel congestion level. The estimated transmission cost in the other node is plotted by Cost*_offload_* using (4), the latency and power consumption weight factors are set as same (i.e., *α* = *β* =1).

If the clock speed is 216 MHz, it is better to directly transfer the raw data when the number of generated packets is 1, which means the channel is idle, whereas when the number generated packets of 30 nodes is more than 2, it is more effective to transmit the data of the output layer. When the number of generated packets is more than 3 and 5, sending the output data of layer 3 and layer 2 is more efficient than transmitting the raw data, respectively. Even when operating at 120 MHz, our proposed offload and transmission strategy can improve the transmission efficiency. However, in the case of an ultralow-power and performance IoT edge device with an operating clock up to 80 MHz, such as STM32L486, it is considered difficult to apply the offload concept, because of the increase in the execution time of the deep learning model.

Figure 6d presents the transmission success ratio of an application data frame without any retransmission. An application data frame consists of several packets; we considered a transmission a failure if one of the packets was lost. The output vector of each layer of the deep learning model should be handled as an application data frame, and all fragmented packets should be successfully transmitted. As shown Figure 6d, in order to increase the transmission success ratio, reducing the number of packets is most important. For example, the output of layer 3 of the applied deep learning model is generated in nine packets; when 30 nodes transmit nine packets within 1 s (i.e., the packet generation time in the simulation), the transmission success ratio is only 12.6% where all nine packets are successfully transmitted in the application layer. However, the transmission success ratio is increased to 99.4% owing to reducing the number of packets by offloading. 

The low-rate wireless personal area network (LR-WPAN) protocol and a low-power MCU were used for the experiment. In addition, we set the MCU to operate in Run mode without any wakeup scheduling from Sleep and Standby mode and set the radio frequency (RF) transceiver to send with low transmission power. Thus, the influence of the current consumption of the MCU and the low transmission power was small in calculating the offload and transmission cost (4), whereas the influence of the execution time for matrix multiplication and the network latency was high. The type of deep learning structures, processors, RF transceivers, and network protocols could significantly impact to the offload and transmission cost.

## 6. Discussion and Conclusions

This paper presented a learning-based MAC protocol with an adaptive channel access scheme using Q-learning to update the Q-values according to channel states and an offload and transmission strategy based on the execution time and the number of generated packets on IoT edge devices. Experiments have shown that our proposed learning-based MAC protocol can improve the transmission success ratio and reduce the latency by an effective channel access method based on optimal backoff time considering the channel congestion levels. Although we have not considered various deep learning structures and experimental environments, the proposed methods can be widely applied because the IoT edge device itself determines whether or not to perform the offload function by considering its computation performance and the number of generated packets according to the current media channel environment.

In the future, we would need to focus on researching how to effectively embed various deep learning structures into ultralow-power and performance MCUs. We are considering applying optimization techniques to quantize the weight parameter to less than 8 bits, such as 3-bit or binary quantization [33,34] and to prune the deep learning network. 

Finally, we hope that the contributions of this study will be to motivate researchers to apply a novel approach for optimizing and offloading IoT edge devices and networks.

## Figures and Tables

**Table 1:** Simulation Parameters for IEEE 802.15.4 with Q-Learning.

Simulation Parameters			
Parameter	Value	Parameter	Value
Simulation time	12 h	Tx power	3 dBm
Network topology	Star	Bit rate	250 kbps
The number of nodes	30 ea	maxMinBE	3
MAC PDU size	112 Bytes	macMaxBE	8
Channel state level		macMaxCSMABackoffs	5
# of pkt < 3: idle,		macMaxRetries	0
3 <= # of pkt < 6: low,		Packet generation	Uniform (0 s, 1 s)
6 <= # of pkt < 9: high,		Send interval	10 s
9 <= # of pkt <= 12: burst		The number of packets	Up to 12 pkt

**Table 2:** Layer parameters and performance for the applied deep learning model.

Model Structure		Input	Kernel		Output Vectors	Parameters	Operations	Data Size (Bytes), # of Fragmented Packets by PDU Size	Execution Time (ms)	
		Dimension (H × W × D)	Stride/Padding	Dimension (H × W × D)	Dimension (H × W × D)				216 MHz	80 MHz
Input		32 × 32 × 3						3072 B, 27 pkts	55	301
CNN Layer 1	Conv 1	32 × 32 × 3	1/2	5 × 5 × 32	32 × 32 × 32	2432	4.9 M	32,768 B, 283 pkts	55	301
	Pool 1	32 × 32 × 32	2/0	3 × 3	16 × 16 × 32		73.7 K	8192 B, 71 pkts	3	14
	Relu 1	16 × 16 × 32			16 × 16 × 32			8192 B, 71 pkts	<1	<1
CNN Layer 2	Conv 1	16 × 16 × 32	1/2	5 × 5 × 32	16 × 16 × 32	25,632	13.1 M	8192 B, 71 pkts	79	427
	Relu 1	16 × 16 × 32			16 × 16 × 32			8192 B, 71 pkts	< 1	1
	Pool 1	16 × 16 × 32	2/0	3 × 3	8 × 8 × 32		18.4 K	2048 B, 18 pkts	1	4
CNN Layer 3	Conv 1	8 × 8 × 32	1/2	5 × 5 × 32	8 × 8 × 64	51,264	6.6 M	4096 B, 36 pkts	39	212
	Relu 1	8 × 8 × 64			8 × 8 × 64			4096 B, 36 pkts	< 1	1
	Pool 1	8 × 8 × 64	2/0	3 × 3	4 × 4 × 64		9.2 K	1024 B, 9 pkts	< 1	1
Fully Connected & Softmax Output Layer 4		4 × 4 × 64			10	10,240	20 K	10 B, 1 pkts	<1 Total: 178	<1 Total: 962
